# *In vitro* effects of *Melaleuca alternifolia* essential oil on growth and production of volatile sulphur compounds by oral bacteria

**DOI:** 10.1590/1678-775720160044

**Published:** 2016

**Authors:** Talita Signoreti GRAZIANO, Caroline Morini CALIL, Adilson SARTORATTO, Gilson César Nobre FRANCO, Francisco Carlos GROPPO, Karina COGO-MÜLLER

**Affiliations:** 1 - Universidade Estadual de Campinas, Faculdade de Odontologia de Piracicaba, Área de Microbiologia e Imunologia, Departamento de Diagnóstico Oral, Piracicaba, SP, Brasil.; 2 - Centro de Diagnóstico de Halitose – Halicenter, São Paulo, SP, Brasil.; 3 - Universidade Estadual de Campinas, Centro Pluridisciplinar de Pesquisas Químicas, Biológicas e Agrícolas, Campinas, SP, Brasil.; 4 - Universidade Estadual de Campinas, Faculdade de Odontologia de Piracicaba, Área de Farmacologia, Anestesiologia e Terapêutica, Departamento de Ciências Fisiológicas, Piracicaba, SP, Brasil.; 5 - Universidade Estadual de Ponta Grossa, Departamento de Biologia Geral, Laboratório de Fisiologia e Patofisiologia, Ponta Grossa, PR, Brasil.; 6 - Universidade Estadual de Campinas, Faculdade de Ciências Farmacêuticas, Campinas, SP, Brasil.

**Keywords:** Products with antimicrobial action, Halitosis. Natural products, P*orphyromonas gingivalis*, Porphyromonas endodontalis

## Abstract

**Objective:**

Halitosis can be caused by microorganisms that produce volatile sulphur compounds (VSCs), which colonize the surface of the tongue and subgingival sites. Studies have reported that the use of natural products can reduce the bacterial load and, consequently, the development of halitosis. The aim of this study was to evaluate the antimicrobial activity of the essential oil of *Melaleuca alternifolia* on the growth and volatile sulphur compound (VSC) production of oral bacteria compared with chlorhexidine.

**Material and Methods:**

The effects of these substances were evaluated by the Minimum Inhibitory Concentration (MIC) and Minimum Bactericidal Concentration (MBC) in planktonic cultures of *Porphyromonas gingivalis* and *Porphyromonas endodontalis*. In addition, gas chromatography analyses were performed to measure the concentration of VSCs from bacterial cultures and to characterize *M. alternifolia* oil components.

**Results:**

The MIC and MBC values were as follows: *M. alternifolia* - *P. gingivalis* (MIC and MBC=0.007%), *P. endodontalis* (MIC and MBC=0.007%=0.5%); chlorhexidine - *P. gingivalis* and *P. endodontalis* (MIC and MBC=1.5 mg/mL). *M. alternifolia* significantly reduced the growth and production of hydrogen sulfide (H_2_S) by *P. gingivalis* (p<0.05, ANOVA-Dunnet) and the H_2_S and methyl mercaptan (CH_3_SH) levels of *P. endodontalis* (p<0.05, ANOVA-Dunnet). Chlorhexidine reduced the growth of both microorganisms without altering the production of VSC in *P. endodontalis*. For *P. gingivalis*, the production of H_2_S and CH_3_SH decreased (p<0.05, ANOVA-Dunnet).

**Conclusion:**

*M. alternifolia* can reduce bacterial growth and VSCs production and could be used as an alternative to chlorhexidine.

## INTRODUCTION

Halitosis, also known as bad breath or malodour, is a condition caused by fetid odours present in air emanating from the mouth^1^, leading to personal and social discomfort^21^. The origin of pathological halitosis can be systemic or local^14^ and should be diagnosed and treated^1^ ^,^ ^14^ ^,^ ^21^. This condition is multifactorial and may comprise both oral and non-oral causes^21^ ^,^ ^25^. Periodontal disease, peri-implantitis, deep carious lesions, tongue coating, impacted food or debris, unclean dentures, and other oral problems may contribute to the onset of halitosis^9^.

Oral bad breath can result from the degradation of proteins containing sulphur amino acids (methionine and cysteine), resulting in the production of volatile sulphur compounds (VSCs), represented by hydrogen sulfide (H_2_S), methyl mercaptan (CH_3_SH), and dimethyl sulphide [(CH_3_)_2_S]: gases that emanate malodour^1^. Some anaerobic gram-negative bacteria present in the oral cavity, such as *Porphyromonas gingivalis, Fusobacterium nucleatum*, *Prevotella intermedia*, *Tannerella forsythia*, and *Porphyromonas endodontalis,* are the main species responsible for the production of VSCs^18^. In addition to the role of VSCs in generating halitosis, there is evidence suggesting that these gases are also involved in the pathogenesis of periodontal diseases^1^ ^,^ ^29^.

Various oral approaches have been employed to treat halitosis, including the mechanical removal of tongue and subgingival biofilms, the use of chlorhexidine, cetylpyridinium, or essential oil mouthrinses, and the application of masking products such as chewing gums and mouthrinses containing chlorine dioxide and zinc salts^5^ ^,^ ^10^ ^,^ ^23^ ^,^ ^26^. It has also been reported that natural products, such as green tea, produce effects that control halitosis and VSC production^12^ ^,^ ^28^ ^,^ ^30^. Most products used to reduce malodour have antimicrobial properties, and the decrease in VSCs is usually related to the suppression of bacterial growth.

*Melaleuca alternifolia,* also known as tea tree oil, has been studied because of its antimicrobial activity against oral pathogens, showing inhibitory and bactericidal effects^3^ ^,^ ^7^ ^,^ ^8^. A solution containing tea tree oil was shown to reduce the levels of malodour and production of VSCs in patients nursed in an intensive care unit^10^. Despite the antimicrobial potential of *M. alternifolia*, there are few studies evaluating its activity against oral pathogens that cause bad breath^4^ ^,^ ^8^. Thus, the aim of this study was to evaluate the effects of *Melaleuca alternifolia* oil and chlorhexidine on the viability and VSC production of *P. gingivalis* and *P. endodontalis*.

## MATERIAL AND METHODS

### Substances tested

This study used the essential oil of *Melaleuca alternifolia* (Arista Industries; Wilton, Connecticut, USA) as the tested substance and chlorhexidine gluconate (Sigma-Aldrich; St. Louis, Missouri, USA) as the standard antimicrobial.

### Determination of the chemical profile of *M. alternifolia* essential oil by gas chromatography-mass spectrometry (GC-MS)

The essential oil was subjected to gas chromatography analyses to obtain its chemical profile. Analyses were performed on a gas chromatograph, model: HP-6890 (HP; Palo Alto, California, USA), interfaced with a mass selective detector HP-5975. A fused silica capillary column HP-5 (length of 30 m, internal diameter of 0.25 mm and film thickness of 0.25 mµ) was used with helium as the carrier gas (1 mL min^-1^).

The oil was diluted with ethyl acetate, and 0.1 mL was injected into the device. The temperatures used were 220°C for the injector, 250°C for the detector, and 60°C – 240°C for the column (3°C min^-1^). To identify the analytes, a mixture of n-alkanes was used to calculate the retention index (RI). Comparisons were performed using the National Institute of Standards and Technology (NIST) electronic library and literature data based on RI. The determination of essential oil components was based on the calculation of the area under the peaks.

### Bacterial strains and culture conditions

*P. gingivalis* W83 and *P. endodontalis* (isolated from clinical sample) were cultivated in Tryptic Soy Broth (TSB – Difco Co.; Detroit, Michigan, USA) or TSA (Tryptic Soy Agar - Difco Co.; Detroit, Michigan, USA), both supplemented with hemin (5 µg/mL), menadione (1 µg/mL), and 2% of Yeast Extract (Difco Co.; Detroit, Michigan, USA). Growth and cultivation were performed under anaerobic conditions (10% CO_2_, 10% H_2,_ and 80% N_2_) using an anaerobic chamber (MiniMacs Anaerobic Workstation - Don Whitley Scientific; Shipley, West Yorkshire UK) at 37°C.

### Minimum inhibitory concentration (MIC) and minimum bactericidal concentration (MBC)

MIC and MBC were carried out according to Clinical and Laboratory Standards Institute (CLSI)^5^ with some modifications. For the determination of MIC and MBC, serial two-fold dilutions were made for all substances tested. Concentrations ranged from 0.5% (v/v) to 0.002% (v/v) for the essential oil of *M. alternifolia* and from 100 µg/mL (0.01% – p/v) to 0.38 µg/mL (0.0038% – p/v) for the chlorhexidine solution. An inoculum of 40% transmittance, equivalent to approximately 8x10^8^ CFU/mL, was prepared from bacterial cultures on TSA with three days of growth, and 500 µL samples of this bacterial suspension were transferred to tubes containing the test substances in a final volume of 6 mL. Moreover, tubes without tested compounds or bacterial suspension were used as controls. Cultures were maintained under anaerobic conditions for 48 hours. The lowest concentration of each substance with no bacterial growth was considered the MIC. For MBC determination, 10 µL samples from TSB cultures were transferred to TSA plates and incubated for five days under anaerobic conditions. The lowest concentration with no bacterial growth was considered the MBC.

### *In vitro* production of Volatile Sulphur Compounds (VSCs)

Sub-MIC concentrations of *M. alternifolia* oil and chlorhexidine were tested to evaluate their effects on VSC production and to ensure bacterial growth and gas production. Thus, concentrations tested were as follows: 0.002%, 0.001%, and 0.0005% for *M. alternifolia*; 0.38 µg/mL (0.0038%), 0.19 µg/mL (0.0019%), 0.095 µg/mL (0.00095%), and 0.048 µg/mL (0.00048%) for chlorhexidine digluconate; representing concentrations 4, 8, and 16 times smaller than the MIC (4x <MIC, 8x <MIC, 16x <MIC).

The inoculum was prepared as described in item 2.3. To collect gas from inside the tubes, needles (Becton Dickinson Company; Franklin Lakes, New Jersey, USA) were coupled with the covers of the tubes. After 16 hours of growth in anaerobic conditions, 1 mL syringes were attached to the needles, and 0.1 mL of air was aspirated from inside each tube. The volume of air collected was injected into the OralChroma™ (CHM-1, Abilit Corporation; Chuo-ku, Osaka, Japan). After measurement, the device provided the concentrations of hydrogen sulfite (H_2_S), methylmercaptan (CH_3_SH), and dimethylsulfide [(CH_3_)_2_S] in parts *per* billion. The tubes were also subjected to absorbance readings (λ=660 nm) in a Unico 1100 RS spectrophotometer (Unico Inc; Dayton, New Jersey, USA).

### Statistical analysis

All experiments were performed in eight replicates and reproduced at least two times. The Lilliefors test (an adaptation of the Kolmogorov-Smirnoff test) was used to check the normality of data distribution. For VSC data, analysis of variance (ANOVA) was performed, and the difference among groups was verified by Tukey test, using the BioEstat software (version 5.0 – AnalystSoft Inc./CNPQ; Belém, Pará, Brazil). The significance level was set at 5%.

## RESULTS

### Chemical profile of the essential oil of *M. alternifolia*

The characterization of essential oil compounds was performed by comparing retention times and MS/MS mass spectra of each peak with information in the NIST library. The compounds identified and the percentage range for tea tree oil recommended by ISO 4730:2004 are described in Table 1.

**Table 1:** Identification of analytes of *M. alternifolia* essential oil compared with the standard composition recommended by ISO 4730; a) TR – peak retention time (in minutes); b) fraction in percent of total integrated area for the chromatogram

t_R_ (min) ^(a)^	Analytes identified	Relative % ^(b)^	% recommended (ISO 4730:2004)
4.66	alpha-thujene	0.72	---
4.84	alpha-pinene	1.98	1.0-6.0
5.87	beta-pinene	0.51	---
6.23	beta-myrcene	0.59	---
6.63	alpha-phellandrene	0.35	---
7.06	alpha-terpinene	9.13	0.5-13
7.27	p-cymene	2.49	0.5-12
7.47	1,8-cineole (Eucalyptol)	3.42	≤ 15
8.5	gamma-terpinene	19.72	10-28
9.41	terpinolene	3.03	1.5-5.0
13.2	terpinen-4-ol	42.07	≥ 30
13.52	alpha-terpineol	2.88	1.5-8.0
22.19	alpha-gurjunene	0.39	---
22.58	trans-caryophyllene	0.36	---
23.39	aromadendrene	1.33	Trace-7.0
24.24	alloaromadendrene	0.58	---
25.48	cis-beta-guaiene	0.17	---
25.85	alpha-muurolene	0.14	---
26.79	delta-cadinene	1.61	Trace-8.0
27.09	cadina-1,4-diene	0.19	---
29.33	viridiflorol	0.18	Trace- 1.5
29.43	cubeban-11-ol	0.16	---
30.7	1-epi-cubenol	0.21	---
59.58	squalene	5.24	---

### Minimum Inhibitory Concentration (MIC) and Minimum Bactericidal Concentration (MBC)

The MIC and MBC values for *M. alternifolia* oil and chlorhexidine solution are shown in Table 2. The tea tree oil and chlorhexidine digluconate solution were able to inhibit the growth of both strains at low concentrations.

**Table 2:** Values of MIC and MBC for *M. alternifolia* essential oil and for the chlorhexidine digluconate solution

Bacterial Strains	*M. alternifolia*	Chlorhexidine	*M. alternifolia*	Chlorhexidine
*P. gingivalis* W83	0.007 %	1.5 µg/ml (0.00015 %)	0.007 %	1.5 µg/ml (0.00015 %)
*P. endodontalis*	0.007 %	1.5 µg/ml (0.00015 %)	0.5 %	1.5 µg/ml (0.00015 %)

### *In vitro* production of Volatile Sulphur Compounds

**Figure 1:**
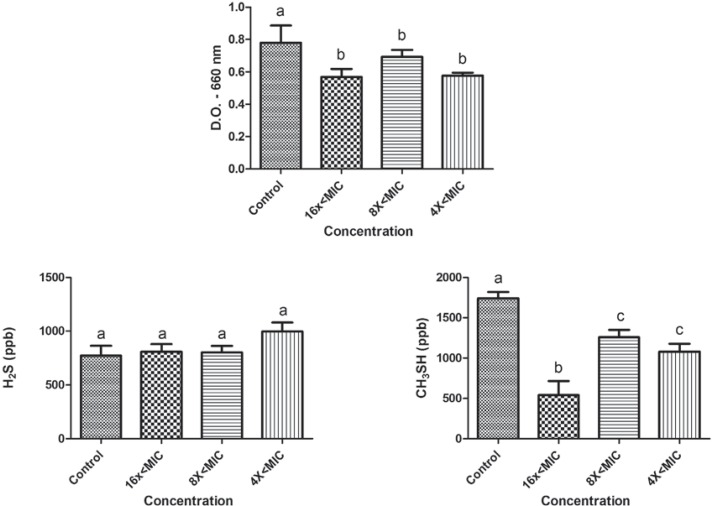
Effects of sub-MIC concentrations of tea tree oil on growth (D.O. – 660 nm) and production of volatile sulphur gases (H2S and CH3SH) for *P. gingivalis* W83. Significant differences among treatments and the control group were considered when p<0.05 (ANOVA, Tukey test). Different letters represent differences among groups

**Figure 2:**
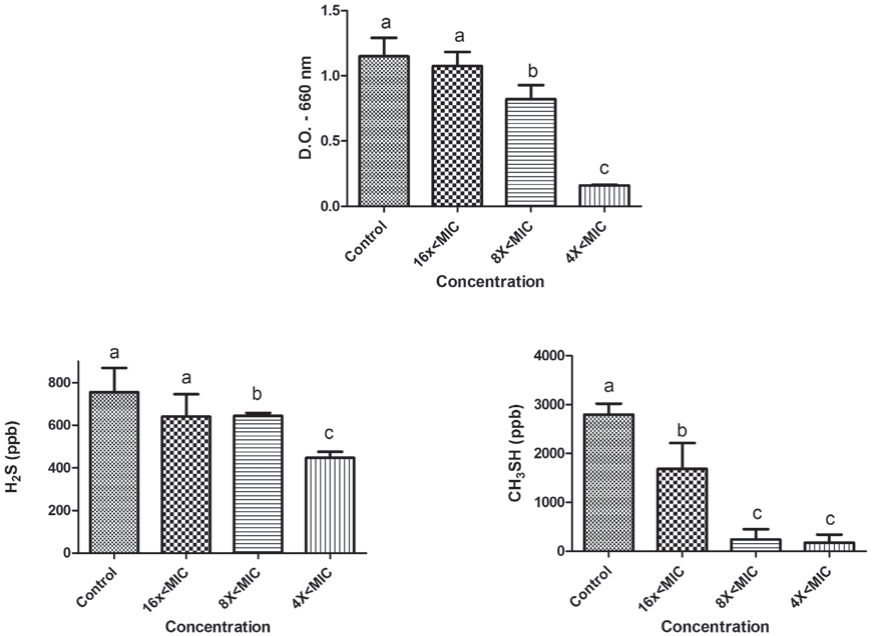
Effects of sub-MIC concentrations of chlorhexidine on growth (D.O. – 660 nm) and production of volatile sulphur gases (H2S and CH3SH) for *P. gingivalis* W83. Significant differences among treatments and the control group were considered when p<0.05 (ANOVA, Tukey test). Different letters represent differences among groups

**Figure 3:**
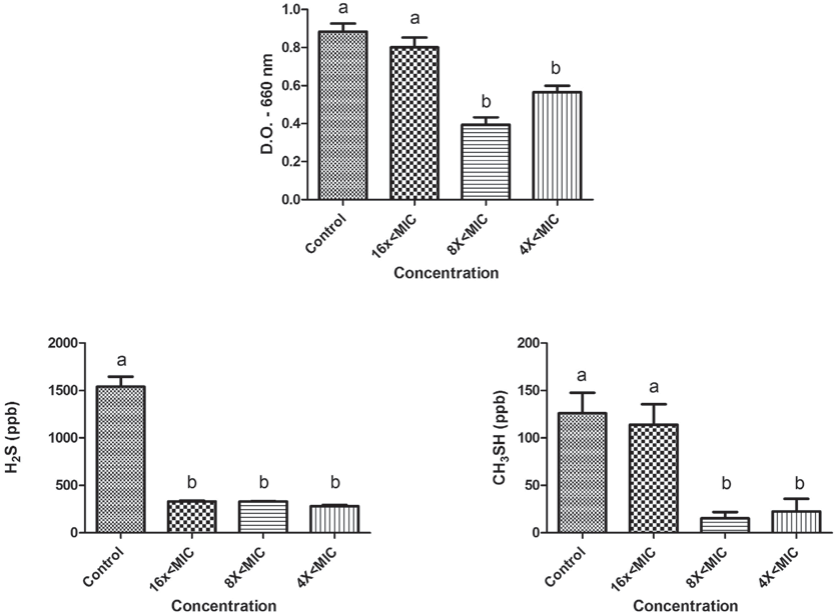
Effects of sub-MIC concentrations of tea tree oil on growth (D.O. – 660 nm) and production of volatile sulphur gases (H2S and CH3SH) for *P. endodontalis*. Significant differences among treatments and the control group were considered when p<0.05 (ANOVA, Tukey test). Different letters represent differences among groups

**Figure 4:**
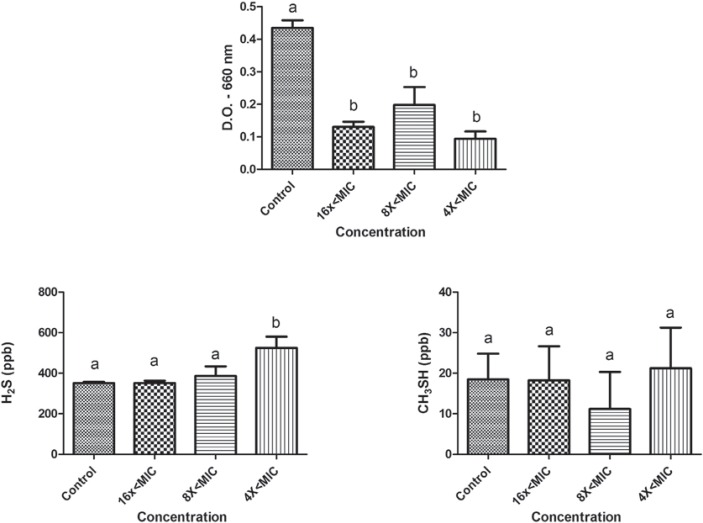
Effects of sub-MIC concentrations of chlorhexidine on growth (D.O. – 660 nm) and production of volatile sulphur gases (H2S and CH3SH) for *P. endodontalis*. Significant differences among treatments and the control group were considered when p<0.05 (ANOVA, Tukey test). Different letters represent differences among groups

The effects of sub-MIC concentrations of tea tree oil and chlorhexidine on the growth and production of volatile sulphur gases (H_2_S and CH_3_SH) are shown in figures 1 and 2 for *P. gingivalis* W83, and in Figures 3 and 4 for *P. endodontalis*. Concentrations tested were lower than the minimum inhibitory concentration (sub-MIC concentrations) and were defined in previous tests.

The tea tree oil reduced the growth of *P. gingivalis* W83 and significantly changed the production of CH_3_SH at all concentrations tested (16x<MIC, 8x<MIC, and 4x<MIC). Curiously, the 16x<MIC concentration promoted less CH_3_SH production than higher concentrations (p<0.05). The production of H_2_S was not altered by the essential oil at any of the concentrations. In the presence of chlorhexidine, *P. gingivalis* W83 showed a reduction in growth at concentrations of 8x<MIC and 4x<MIC, and they were different from each other (p<0.05). Furthermore, it exhibited a reduction in CH_3_SH levels for all concentrations tested and in H_2_S for 8x<MIC and 4x<MIC (p<0.05).

The microorganism *P. endodontalis* showed a reduction in growth in the presence of tea tree oil for concentrations 8x<MIC and 4x<MIC (p<0.05). Decreased gas production was observed for both H_2_S (16x<MIC, 8x<MIC, and 4x<MIC) and CH_3_SH (8x<MIC and 4x<MIC) (p<0.05). However, at the concentrations tested, chlorhexidine showed effects on *P. endodontalis* growth (p<0.05) but not on VSC production.

## DISCUSSION

As a result of the significant contribution of VSCs to halitosis development, many studies involving therapies for the treatment of halitosis have focused on the inhibition and reduction of VSC production. Antimicrobial therapy aims to enhance mechanical treatments and support host defences, reducing the development of microorganisms. Some studies have demonstrated the efficacy of *M. alternifolia* against oral bacteria^7^ ^,^ ^8^ ^,^ ^11^ ^,^ ^24^; however, little is known about its effects on VSC production. Here, we showed that *M. alternifolia* essential oil can reduce growth and VSC levels of *P. gingivalis* and *P. endodontalis*, even at sub-MIC concentrations.

GC-MS analysis of the tea tree oil used in this study showed that the composition of this oil was consistent with the International Standard ISO 4730:2004, which specifies certain characteristics of *M. alternifolia* oil such as quality requirements. The main components described by the ISO for *M. alternifolia* oil are terpinen-4-ol, γ-terpinene, α-terpinene, 1,8-cineole, p-cymene, α-terpineol, α-pinene, terpinolene, limonene, and sabinene^20^. All of these compounds were found in the oil used in this study, except for limonene and sabinene. However, these two compounds are generally found in small quantities in tea tree oil (0.5% - 4% and trace – 3.5%, respectively), and their low levels may be the reason they were not identified by CG-MS analysis. In contrast, terpinen-4-ol and α-terpineol, which are substances with antimicrobial activity, were found in satisfactory percentages^4^ ^,^ ^13^. *M. alternifolia* have a mixture of components and their mechanisms of action are not completed elucidated. It is known that the combination of these different substances in the tea tree oil are capable of inducing loss of intracellular material, inhibition of respiration, and alterations in the homeostasis, leading to loss of bacterial membrane integrity and function^4^.

The tea tree oil showed antimicrobial activity: its MIC value was 0.007% for both bacteria, and MBC values were 0.5% for *P. endodontalis* and 0.007% for *P. gingivalis*. In a previous study, the MIC value found for *P. gingivalis* was 0.13% - 0.25%, and the MBC value was 0.13% - 0.5%^22^. Although MIC values differed between studies, MBC values were similar. Differences in MIC values may be due to different strains tested: in this study we used the W83 strain, while Takarada, et al.^22^ (2004) used the ATCC 33277, 53977, Su63, and W50 strains. To the best of our knowledge, there are no previous studies in the literature showing the effects of *M. alternifolia* on *P. endodontalis*.

To evaluate the activity of *M. alternifolia* and chlorhexidine on the production of volatile sulphur compounds, sub-inhibitory concentrations of these substances were used. The sub-MIC concentrations of the tea tree oil affected the growth of both microorganisms, reducing the growth at higher concentrations (8x<, 4x<MIC, and 16x<MIC). Furthermore, at all concentrations tested, tea tree oil reduced CH_3_SH production in *P. gingivalis* and both H_2_S and CH_3_SH production in *P. endodontalis*. As not expected, the 16x<MIC concentration promoted a higher reduction of CH_3_SH than the 8x< and 4x<MIC concentrations. We believe that higher concentrations of tea tree oil (8x and 4x<MIC) could stress the bacteria and stimulate a little the VSC production comparing to the lower concentrations (16x<MIC). However, this hypothesis has to be confirmed.

The CH_3_SH and H_2_S gases are the main VSCs in intra-oral halitosis^1^, and their toxicity is associated with the development of periodontal disease^15^ ^,^ ^16^ ^,^ ^29^. Tea tree oil was able to inhibit the growth of *P. gingivalis* W83 and *P. endodontalis*, and the production of VSCs. Thus, this essential oil is a promising substance for treating halitosis. Previously, *M. alternifolia* oil was found to be effective as one of the components of an antiseptic mouth rinse^10^. In addition, α-terpineol, one of the compounds present in tea tree oil and identified in our study, has demonstrated activity against a number of oral pathogens involved in periodontal disease and caries^17^.

Chlorhexidine is widely used in mouthrinses, causing membrane disruption and inhibition of proteolytic and glycosidic enzimes, leading to growth inhibition and cell death^27^. Despite being a potent antimicrobial, it has certain side effects such as altered taste, mucosal desquamation, tooth staining, increased calcified supra-gingival deposits, and a burning sensation in the oral mucosa^19^. Compared with chlorhexidine, tea tree oil showed similar antimicrobial activity, promoting bactericidal and bacteriostatic effects at low concentrations. Thus, tea tree oil could be a good alternative to chlorhexidine in oral hygiene products, mainly mouthrinses. Therefore, the development of new mouthrinses containing tea tree oil and clinical studies testing these products are necessary.

In conclusion, *M. alternifolia* oil showed antimicrobial activity against *P. gingivalis* W83 and *P. endodontalis*, reducing the growth and the production of VSCs at sub-MIC concentrations, comparably to chlorhexidine. Future studies can be conducted focusing on the development of pharmaceutical products containing *M. alternifolia* oil for halitosis treatment.

## References

[1] Akaji EA, Folaranmi N, Ashiwaju O (2014). Halitosis: a review of the literature on its prevalence, impact and control. Oral Heal Prev Dent.

[2] Aylıkcı BU, Colak H (2013). Halitosis: from diagnosis to management. J Nat Sci Biol Med.

[3] Campos Rasteiro VM, Costa AC, Araújo CF, Barros PP, Rossoni RD, Anbinder AL (2014). Essential oil of Melaleuca alternifolia for the treatment of oral candidiasis induced in an immunosuppressed mouse model. BMC Complement Altern Med.

[4] Carson CF, Riley TV (1995). Antimicrobial activity of the major components of the essential oil of Melaleuca alternifolia. J Appl Bacteriol.

[5] Clinical and Laboratory Standards Institute – CLSI (2007). Methods for dilution antimicrobial susceptibility tests for bacteria that grow anaerobically. M11-A7.

[6] Erovic Ademovski S, Lingström P, Winkel E, Tangerman A, Persson GR, Renvert S (2012). Comparison of different treatment modalities for oral halitosis. Acta Odontol Scand.

[7] Groppo FC, Ramacciato JC, Simões RP, Flório FM, Sartoratto A (2002). Antimicrobial activity of garlic, tea tree oil, and chlorhexidine against oral microorganisms. Int Dent J.

[8] Hammer KA, Dry L, Johnson M, Michalak EM, Carson CF, Riley TV (2003). Susceptibility of oral bacteria to Melaleuca alternifolia (tea tree) oil in vitro. Oral Microbiol Immunol.

[9] Hinode D, Fukui M, Yokoyama N, Yokoyama M, Yoshioka M, Nakamura R (2003). Relationship between tongue coating and secretory-immunoglobulin A level in saliva obtained from patients complaining of oral malodor. J Clin Periodontol.

[10] Hur MH, Park J, Maddock-Jennings W, Kim DO, Lee MS (2007). Reduction of mouth malodour and volatile sulphur compounds in intensive care patients using an essential oil mouthwash. Phytother Res.

[11] Karbach J, Ebenezer S, Warnke PH, Behrens E, Al-Nawas B (2015). Antimicrobial effect of Australian antibacterial essential oils as alternative to common antiseptic solutions against clinically relevant oral pathogens. Clin Lab.

[12] Lodhia P, Yaegaki K, Khakbaznejad A, Imai T, Sato T, Tanaka T (2008). Effect of green tea on volatile sulfur compounds in mouth air. J Nutr Sci Vitaminol (Tokyo).

[13] Loughlin R, Gilmore BF, McCarron PA, Tunney MM (2008). Comparison of the cidal activity of tea tree oil and terpinen-4-ol against clinical bacterial skin isolates and human fibroblast cells. Lett Appl Microbiol.

[14] Luqman M (2012). Systemic origin of halitosis : a review. Int J Clin Dent Sci.

[15] Makino Y, Yamaga T, Yoshihara A, Nohno K, Miyazaki H (2012). Association between volatile sulfur compounds and periodontal disease progression in elderly non-smokers. J Periodontol.

[16] Nakano Y, Yoshimura M, Koga T (2002). Correlation between oral malodor and periodontal bacteria. Microbes Infect.

[17] Park SN, Lim YK, Freire MO, Cho E, Jin D, Kook JK (2012). Antimicrobial effect of linalool and α-terpineol against periodontopathic and cariogenic bacteria. Anaerobe.

[18] Persson S, Claesson R, Carlsson J (1989). The capacity of subgingival microbiotas to produce volatile sulfur compounds in human serum. Oral Microbiol Immunol.

[19] Prayitno S, Addy M (1979). An in vitro study of factors affecting the development of staining associated with the use of chlorhexidine. J Periodontal Res.

[20] Sciarrone D, Ragonese C, Carnovale C, Piperno A, Dugo P, Dugo G (2010). Evaluation of tea tree oil quality and ascaridole: a deep study by means of chiral and multi heart-cuts multidimensional gas chromatography system coupled to mass spectrometry detection. J Chromatogr A.

[21] Seemann R, Duarte da Conceição M, Filippi A, Greenman J, Lenton P, Nachnani S (2014). Halitosis management by the general dental practitioner - results of an International Consensus Workshop. Swiss Dent J.

[22] Takarada K, Kimizuka R, Takahashi N, Honma K, Okuda K, Kato T (2004). A comparison of the antibacterial efficacies of essential oils against oral pathogens. Oral Microbiol Immunol.

[23] Thaweboon S, Thaweboon B (2011). Effect of an essential oil-containing mouth rinse on VSC-producing bacteria on the tongue. Southeast Asian J Trop Med Public Heal.

[24] Thosar N, Basak S, Bahadure RN, Rajurkar M (2013). Antimicrobial efficacy of five essential oils against oral pathogens: an in vitro study. Eur J Dent.

[25] Van den Broek A, Feenstra L, de Baat C (2008). A review of the current literature on management of halitosis. Oral Dis.

[26] Van Leeuwen MP, Slot DE, Van der Weijden GA (2011). Essential oils compared to chlorhexidine with respect to plaque and parameters of gingival inflammation: a systematic review. J Periodontol.

[27] Van Leeuwen MP, Slot DE, Van der Weijden GA (2014). The effect of an essential-oils mouthrinse as compared to a vehicle solution on plaque and gingival inflammation: a systematic review and meta-analysis. Int J Dent Hyg.

[28] Xu X, Zhou XD, Wu CD (2010). Tea catechin EGCg suppresses the mgl gene associated with halitosis. J Dent Res.

[29] Yaegaki K, Qian W, Murata T, Imai T, Sato T, Tanaka T (2008). Oral malodorous compound causes apoptosis and genomic DNA damage in human gingival fibroblasts. J Periodontal Res.

[30] Zeng QC, Wu AZ, Pika J (2010). The effect of green tea extract on the removal of sulfur-containing oral malodor volatiles in vitro and its potential application in chewing gum. J Breath Res.

